# Myricetin Prevents Alveolar Bone Loss in an Experimental Ovariectomized Mouse Model of Periodontitis

**DOI:** 10.3390/ijms17030422

**Published:** 2016-03-22

**Authors:** Jialiang Huang, Chuanlong Wu, Bo Tian, Xiao Zhou, Nian Ma, Yufen Qian

**Affiliations:** 1 Department of Orthodontics, Shanghai Ninth People’s Hospital, Shanghai Jiao Tong University, School of Medicine, 639 Zhizaoju Road, Shanghai 200011, China; huangjialiang1985@163.com (J.H.); xzhouaa@163.com (X.Z.); nianmaaa@163.com (N.M.); 2 Shanghai Key Laboratory of Orthopaedic Implants, Department of Orthopaedics, Shanghai Ninth People’s Hospital, Shanghai Jiao Tong University, School of Medicine, Shanghai 200011, China; challengewu1988@163.com (C.W.); poetian@163.com (B.T.)

**Keywords:** myricetin, periodontitis, alveolar bone resorption, osteoclast formation

## Abstract

Periodontitis is a common chronic inflammatory disease, which leads to alveolar bone resorption. Healthy and functional alveolar bone, which can support the teeth and enable their movement, is very important for orthodontic treatment. Myricetin inhibited osteoclastogenesis by suppressing the expression of some genes, signaling pathways, and cytokines. This study aimed to investigate the effects of myricetin on alveolar bone loss in an ovariectomized (OVX) mouse model of periodontitis as well as *in vitro* osteoclast formation and bone resorption. Twenty-four healthy eight-week-old C57BL/J6 female mice were assigned randomly to four groups: phosphate-buffered saline (PBS) control (sham) OVX + ligature + PBS (vehicle), and OVX + ligature + low or high (2 or 5 mg∙kg^−1^∙day^−1^, respectively) doses of myricetin. Myricetin or PBS was injected intraperitoneally (i.p.) every other day for 30 days. The maxillae were collected and subjected to further examination, including micro-computed tomography (micro-CT), hematoxylin and eosin (H&E) staining, and tartrate-resistant acid phosphatase (TRAP) staining; a resorption pit assay was also performed *in vitro* to evaluate the effects of myricetin on receptor activator of nuclear factor κ-B ligand (RANKL)-induced osteoclastogenesis. Myricetin, at both high and low doses, prevented alveolar bone resorption and increased alveolar crest height in the mouse model and inhibited osteoclast formation and bone resorption *in vitro*. However, myricetin was more effective at high dose than at low dose. Our study demonstrated that myricetin had a positive effect on alveolar bone resorption in an OVX mouse model of periodontitis and, therefore, may be a potential agent for the treatment of periodontitis and osteoporosis.

## 1. Introduction

Periodontitis is a common chronic inflammatory disease that destroys the supporting structures of the teeth by complex and multifactorial pathogenic processes, which lead to alveolar bone resorption [1,2]. There is increasing evidence that orthodontists are more likely to encounter adult patients with periodontal diseases, especially periodontitis, which are often associated with chronic diseases such as osteoporosis [1,3,4]. Abnormal resorptive activity of the osteoclasts is one of the major causes of periodontitis and osteoporosis [5,6]. Therefore, targeting osteoclastic resorption is a significant therapeutic strategy.

While several drug therapies including bisphosphonates [7] and anti- receptor activator of nuclear factor κ-B ligand (RANKL) denosumab [8] have been successfully used to inhibit osteoclastic activity, these treatments have some severe adverse effects such as gastrointestinal toxicity, osteonecrosis of the jaw, endometrial cancer, and thromboembolism [9,10].

Myricetin (3,3′,4′,5,5′,7-hexahydroxyflavone) is a naturally occurring flavonoid compound, which is widespread and present abundantly in plants such as tea, berries, fruits, vegetables, and medicinal herbs. Studies have shown that myricetin protects osteoblasts against apoptotic cell death induced by inflammatory cytokines and induces osteoblast maturation and differentiation [11,12]. Recent studies have reported that myricetin inhibits osteoclastogenesis and Ti particle-induced osteolysis, indicating it may be potentially useful in the treatment osteoclast-related osteolytic diseases [13,14]. Moreover, a study *in vitro* shows the anti-destructive effects of myricetin on human gingival fibroblasts under inflammatory conditions, and the anti-osteoclastogenetic effects of myricetin on the related receptor activators, which suggest that myricetin may have therapeutic effects in periodontal diseases [15]; however, no studies have been conducted to prove this, and its effect on inhibiting alveolar bone loss are not well understood. In this study, we examined the effects of myricetin on alveolar bone loss (ABL) in mouse model of periodontitis, and the effects of myricetin on osteoclast formation and function were also investigated. We conjecture that myricetin could block ABL, and may have potentially therapeutic effects in the periodontitis and osteoporosis.

Therefore, the present study aims to: (1) investigate the potential therapeutic benefits of myricetin for alveolar bone loss in an experimental ovariectomized (OVX) mouse model of periodontitis; and (2) verify the effects of myricetin on osteoclast formation and bone resorption *in vitro*.

## 2. Results

### 2.1. Micro-Computed Tomography (Micro-CT) Analysis of Alveolar Bone

**Figure 1:**
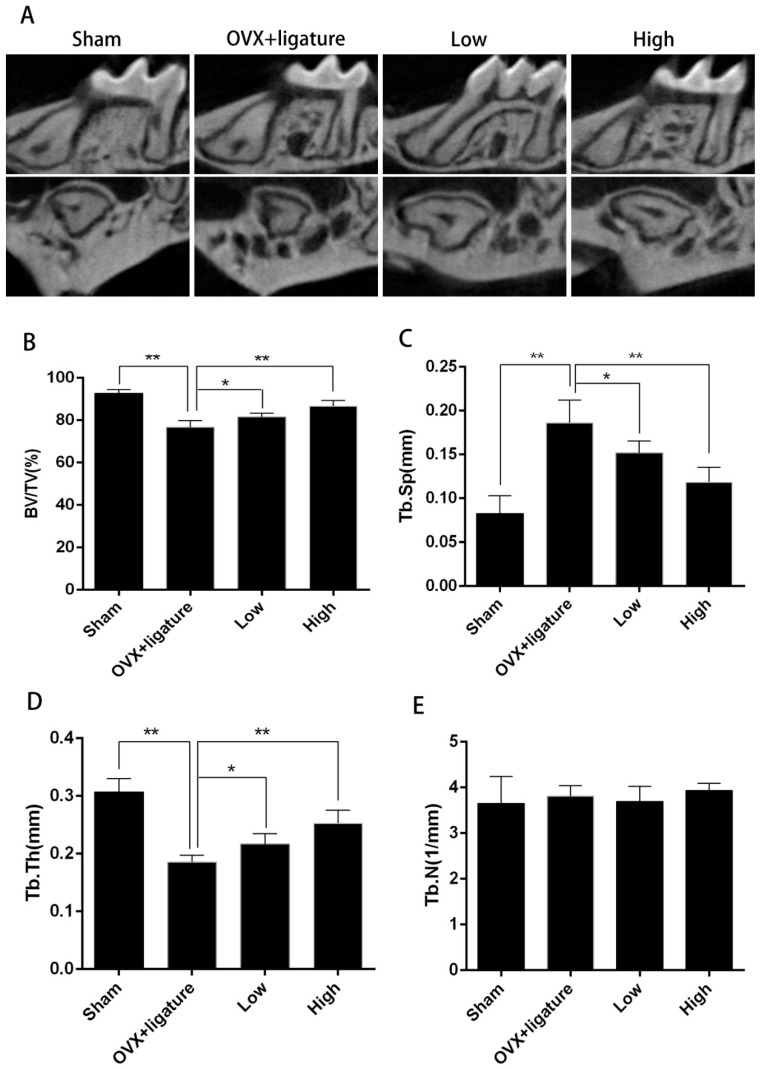
Micro computed tomography (CT) analysis of bone volume showing effect of myricetin on maxilla parameters. (**A**) Longitudinal and transverse-sectional micro-CT images of first molar (M1) in maxilla; (**B**–**E**) Analysis of micro-CT volumetric parameters: bone volume/tissue volume (BV/TV), trabecular separation (Tb.Sp), trabecular thickness (Tb.Th), trabecular number (Th.N), and ovariectomized (OVX); * *p* < 0.05, ** *p* < 0.017; *n* = 6.

The micro-computed tomography (Micro-CT) scanning and 3D reconstruction revealed more obvious alveolar bone resorption of the upper first molar (M1) in the OVX + ligature group than there was in the control (sham). In addition, the bone resorption in mice treated with both high and low doses of myricetin was considerably lower than that in the control (sham) mice (Figure 1A). Compared to mice in the OVX + ligature group, those receiving myricetin at low and high doses (2 and 5 mg∙kg^−1^∙day^−1^, respectively) showed significantly higher bone volume/tissue volume (BV/TV) and trabecular thickness (Tb.Th) and significantly lower trabecular separation (Tb.Sp) (Figure 1B–D). There was no significant difference in trabecular number (Th.N) (Figure 1E) among the groups.

**Figure 2:**
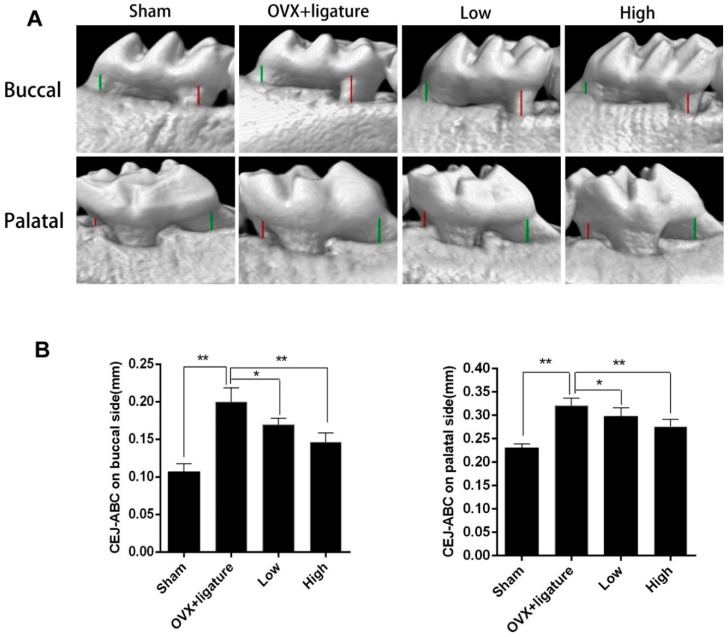
Effect of myricetin on maxilla of cemento-enamel junction (CEJ) to alveolar bone crest (ABC). (**A**) Buccal and palatal sides of maxilla showing alveolar bone loss (ABL, colored lines) was measured from CEJ to ABC at 4 points: mesiobuccal (MB, red), mesiolingual (ML, red), distobuccal (DB, green), and distolingual (DL, green) regions for first maxillary molar (M1); (**B**) Analysis of CEJ-ABC linear distance after myricetin or placebo treatment; * *p* < 0.05, ** *p* < 0.017; *n* = 6.

There was an obvious decrease in the alveolar crest height in the OVX + ligature group (Figure 2A). Compared to the OVX + ligature group, the low- and high-dose myricetin groups partially prevented the reduction in alveolar crest height at the buccal and palatal sides (Figure 2A,B).

### 2.2. Histologcal and Histomorphometric Analysis of Alveolar Bone

**Figure 3:**
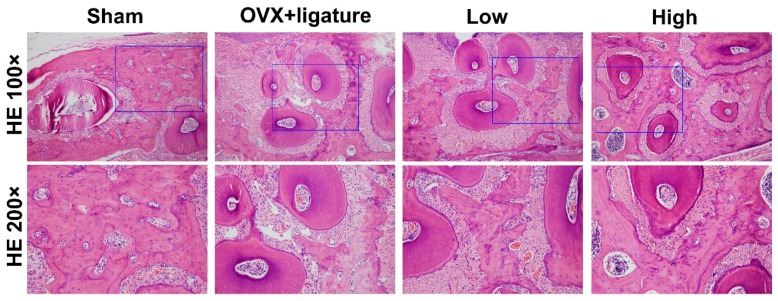
Descriptive analysis of hematoxylin and eosin (H&E) staining in paraffin sections; images show cross sections of each group at 100× and 200× magnification (*n* = 6).

**Figure 4:**
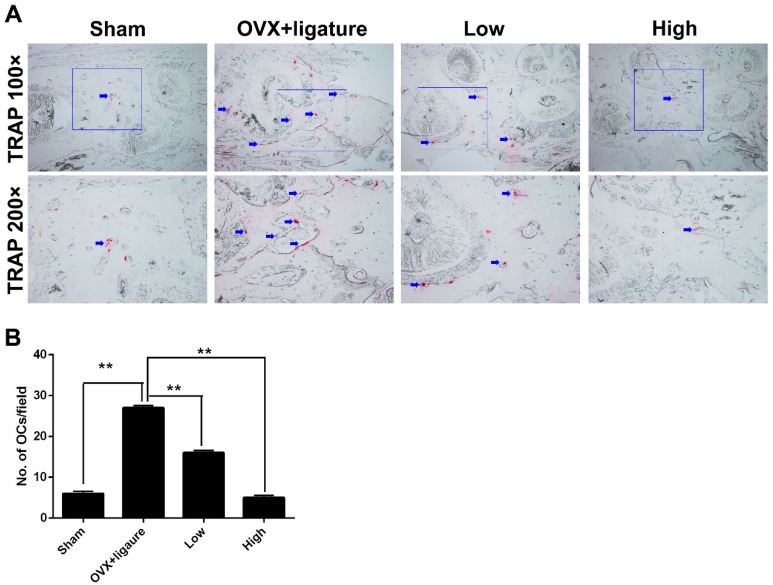
Analysis of tartrate-resistant acid phosphatase (TRAP) staining: (**A**) 100× and 200× magnified images of alveolar bone after TRAP staining and osteoclasts are stained red (blue arrows); and (**B**) number of osteoclasts per field of tissue (No. of OCs/field) in 200× magnification were analyzed; ** *p* < 0.017 between groups (*n* = 6).

Histological and histomorphometric analysis further confirmed that myricetin treatment protected against ABL. The hematoxylin and eosin (H&E) stained sections showed some alveolar bone resorption in the sham group while it had clearly occurred in the OVX + ligature group. However, the myricetin-treated groups exhibited reduced bone resorption, and the high-dose group showing a superior effect (Figure 3). Tartrate-resistant acid phosphatase (TRAP) staining revealed that the number of multinucleated osteoclasts increased and obviously decreased in the OVX + ligature and high-dose myricetin, respectively (Figure 4A,B).

### 2.3. Myricetin Inhibited Receptor Activator of Nuclear Factor κ-B Ligand (RANKL)-Induced Osteoclast Formation without Cytotoxicity

Cell Counting Kit-8 (CCK8) assays were performed to examine the potential cytotoxicity of myricetin. The inhibitory concentration (IC_50_) of myricetin was 551.5 μM at 96 h, indicating that it may inhibit the proliferation of RAW264.7 cells but only at high concentrations (>551.5 μM).

**Figure 5:**
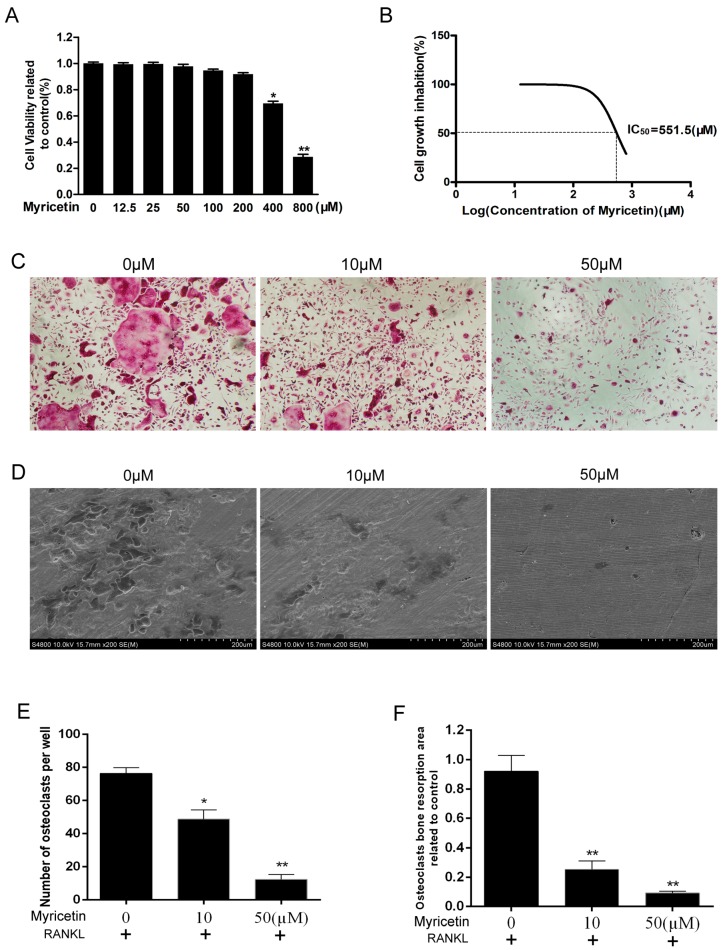
Myricetin inhibited receptor activator of nuclear factor κ-B ligand (RANKL)-induced osteoclastogenesis and osteoclast-mediated bone resorption without cytotoxicity. (**A**) RAW264.7 cells were treated with 50 ng/mL RANKL, and indicated concentrations of myricetin for 96 h prior to measuring cell viability using Cell Counting Kit-8 (CCK8) assay; * *p* < 0.05, ** *p* < 0.017 (*n* = 3); (**B**) Inhibition rate of RAW264.7 cells was calculated (GraphPad Prism), and the half-maximal inhibitory concentration (IC_50_) was determined as 551.5 μM; (**C**) RAW264.7 cells were treated with indicated concentrations of myricetin and 50 ng/mL RANKL, respectively for 5 days. Cells were fixed with 4% (*w*/*v*) paraformaldehyde and stained for TRAP; (**D**) Representative scanning electron microscopy (SEM) images of bone-resorption pits are shown; (**E**) Number of TRAP-positive cells; (**F**) Total area of resorption pits measured using Image J; * *p* < 0.05, ** *p* < 0.017 (*n* = 3).

We investigated the effect of myricetin on osteoclastogenesis by stimulating RAW264.7 cells with RANKL (50 ng/mL), and myricetin (0, 10, and 50 μM) during osteoclast formation. The RAW264.7 cells differentiated into characteristic TRAP-positive multinucleated osteoclast-like (OCL) cells (Figure 5C). However, cells exposed to myricetin showed a significant dose-dependent decrease in the number of TRAP positive OCL cells compared to the control cells. Osteoclast formation was suppressed by approximately 30% by 10 μM myricetin and almost completely at higher concentrations (≥50 μM) (Figure 5E). Myricetin did not affect the proliferation of RAW264.7 cells at either 25 or 50 μM (Figure 5A,B). These results suggest that these concentrations of myricetin impaired osteoclast formation without causing cell death.

### 2.4. Myricetin Attenuated Osteoclastic Bone Resorption

We hypothesized that osteoclast bone resorption would also be inhibited because osteoclast differentiation was obviously impaired by myricetin. We performed an osteoclastic bone resorption assay to evaluate this hypothesis. Scanning electron microscopy (SEM) analysis indicated that the bone surface was resorbed by osteoclasts actively *in vitro*. More than 50% of this bone resorption activity was effectively inhibited by 10 μM myricetin, and almost completely blocked at higher myricetin concentrations (≥50 μM, Figure 5D,F). This result clearly indicated that the administration of myricetin reduced bone resorption *in vitro*.

## 3. Discussion

Surveys of periodontal disease reveal a 15%–20% prevalence of severe periodontitis within the Asian [16] and a 5%–15% prevalence of advanced periodontitis in the global adult population [17]. Osteoporosis may act as a risk factor for periodontal disease with inflammatory loss of alveolar bone [18,19,20]. Favorable alveolar bone, which can support and enable the teeth to move, is very important for orthodontic treatment [21,22]. Periodontitis is a great challenge to orthodontists because of the potential for progression of the disease [23]. Healthy periodontal tissue is also the basis for all successful dental treatment. To date, numerous advances have been made in the treatment of osteolytic diseases using bisphosphonates, estrogens, and teriparatide; however, they are associated with many severe adverse effects [9,10,24]. In this study, we demonstrated that myricetin inhibited alveolar bone resorption *in vivo*, which suggests its potential usefulness in the treatment of patients with osteoporosis and periodontitis.

We developed an OVX mouse model of experimentally induced osteoporosis and periodontitis. OVX animals are widely used as a model of osteoporosis, which is induced by the estrogen deficiency. OVX mice exhibit severe bone loss in the femur and inflammatory cytokines such as interleukin-1 (IL-1), IL-6, and prostaglandin E2 (PGE2) may be involved in the mechanism of bone loss due to estrogen deficiency [25,26,27,28]. These cytokines are also involved in the inflammatory pathogenesis of periodontitis [4]. Kobayashi *et al.* [18] also found that the OVX mouse model showed alveolar bone loss and that estrogen deficiency significantly enhanced this loss of alveolar bone in lipopolysaccharide (LPS)-induced periodontitis. In addition, the anti-bone resorptive drugs used for postmenopausal osteoporosis may also be effective for managing periodontitis accompanied by local alveolar bone loss [18]. This study corroborates previous results showing that the alveolar bone was obviously reduced in OVX mice. Therefore, the OVX mouse model of periodontitis and osteoporosis would be useful for investigating preventative and curative treatments for various diseases affecting the bones and teeth.

The present study identified obvious alveolar bone resorption in the OVX group, whereas the myricetin-treated groups showed a significant and dose-dependent suppression of alveolar bone loss without any obvious adverse effects. The histological analysis of tissues stained with H&E and TRAP further supported these results because they revealed that myricetin reduced inflammation, osteolysis, and the number of TRAP-positive multinucleated osteoclasts. Therefore, the inhibition of osteoclast formation and function, and secretion of inflammatory factors may be the key targets for therapeutic agents in the treatment of OVX-induced periodontitis. The micro-CT measurement showed that both the low- and high-dose myricetin groups increased the BV/TV.

ABL is considered as an indicator of the periodontitis process and reflects the line distance of cemento-enamel junction to alveolar bone crest (CEJ-ABC) [29]. In this study, we demonstrated that the extent of ABL in the OVX group was rather high, which indicated severe bone loss. The OVX mice exhibited severe periodontitis while treatment with myricetin significantly reduced the bone loss and inhibited the periodontitis process, as shown by the H&E and TRAP staining results.

The bone resorption assays revealed that the number and area of bone resorption pits observed *in vitro* dramatically decreased after myricetin treatment. These effects were mediated by the dose-dependent inhibition of osteoclast formation. This inhibition was observed at concentrations of 10 and 50 μM myricetin and occurred without inducing cytotoxicity, which was also previously shown by Wu *et al.* [13]. Osteoclasts play a key role in the alveolar bone resorption associated with periodontitis. Myricetin inhibited the expression of a number of osteoclastogenesis-related genes including *TRAP*, calcitonin receptor (*CTR*), cathepsin K (*CTSK*), vacuolar-type H^+^ *(V-)-ATPase-d2*, *c-fos*, and nuclear factor of activated T-cells, cytoplasmic 1 (*NFATc1*) [13,15]. In addition, it inhibited signaling pathways including p-38, extracellular signal-regulated kinase (ERK), cSrc, mitogen-activated protein kinase (MAPK) and nuclear factor κ-light-chain-enhancer of activated B cells (NF-κB) as well as cytokines such as tumor necrosis factor-α (TNF-α) and IL-1β, which are all related to osteoclastogenesis [13,15]. Furthermore, a previous study [15] also showed that myricetin decreased the mRNA expression and enzyme activity of matrix metalloproteinase-1 (MMP-1), MMP-2, and MMP-8, which were involved in the inflammatory progression in periodontitis in the human gingival fibroblasts (HGF). This may explain why myricetin reduced alveolar bone resorption and showed therapeutic effects in periodontal diseases.

Over the last two decades, numerous advances have been made in the treatment of periodontitis. (1) Nonsurgical therapy such as scaling and root planing using both hand and ultrasonic instruments are effective in treating periodontitis [30]. Furthermore, aggressive periodontitis responds well to short-term scaling and root planing (up to six months); however, relapse and disease progression have been reported after six months despite frequent recall visits and oral-hygiene reinforcements [31]; (2) Surgical treatments such as modified Widman flap surgery, bone grafting, and guided tissue regeneration using membranes provide the practitioner with direct access to root surfaces and furcation areas, which permits a more thorough debridement to facilitate regeneration of defective bone [32,33,34,35,36]; (3) Systemic antibiotics and local antimicrobials such as tetracycline have been reported to decreased the probing pocket depths and resulted in development of clinical attachment for up to at least 24 months [37,38,39].

The use of myricetin in the clinical treatment of periodontitis would be enhanced by its local application at the target site and combined with surgical treatments. This may enhance the convenience and practicality of using myricetin since it plays a more direct role locally than it does systemically. Xu *et al.* [29] found that local and oral administration of simvastatin had a positive effect on OVX rats with experimentally induced periodontitis. Slots and Rosling [40] administered tetracycline for two weeks after the completion of an initial phase of scaling and root planing. Unlike antibiotics, local application of myricetin, which inhibits the osteoclast formation, may be more effective in treating patients with osteoporosis who have periodontitis.

An increasing number of adults are seeking orthodontic treatment for various reasons, and compared to children, some may additionally have periodontitis and osteoporosis. Orthodontic forces cause bone resorption, which is controlled by the rate of osteoclast formation. Therefore, myricetin may reduce tooth movement. The use of myricetin in patients receiving orthodontic treatment requires the consideration of its therapeutic half-life. Further experiments are necessary to explore the movement of teeth after myricetin application.

In addition, there are other limitations of this study. First, there is only one experiment time in this study; drug onset time, the impact and side effects of long-term medication are unclear. We have attached great importance to set the experimental period. Referring to the methods described by Wu *et al.* [13], both low and high (2 or 5 mg∙kg^−1^∙day^−1^, respectively) dose myricetin, which were injected every other day for 14 days, have inhibitory bone loss in mouse model. According to the methods of Xu *et al.* [29], simvastatin, which was given (local or oral) for two months, could prevent alveolar bone loss in rat model of periodontitis. Thus, we set this doses and experimental period mentioned above. The results were satisfactory, and no adverse effects or mortality occurred during the experiment; Secondly, ovariectomy will cause systemic osteoporosis, which may affect the results of the study. Except ovariectomized case, a nylon ligature placed around the cervix of molars in rat could also induce periodontitis [41], so, we may do further research to explore the effect of myricetin in this model; Finally, myricetin may protect osteoblasts against apoptotic cell death induced by inflammatory cytokines and induces osteoblast maturation and differentiation [11,12]. In this study, we mainly examine the effects of myricetin on ABL in mouse model of periodontitis, and mechanism caused by the abnormal osteoclast formation and function. We will involve aspects of osteoblasts in the next study.

There are numerous strengths to this study. For instance, to the best of our knowledge, this is the first study to characterize the inhibitory effects of myricetin on alveolar bone resorption. Compared to synthetic drugs, Chinese herbs have fewer side effects and are less prone to drug resistance. Therefore, further investigation of the effects of myricetin is a promising approach in the direction of developing novel treatments for periodontitis.

## 4. Materials and Methods

### 4.1. Media and Reagents

The media and reagents used in the experiments and their sources are as follows: Mouse macrophage RAW264.7 cells (American Type Culture Collection, ATCC, Rockville, MD, USA). α-modification of Eagle’s medium (α-MEM) and fetal bovine serum (FBS) (Gibco-BRL, Sydney, Australia). Myricetin (Sigma-Aldrich, St. Louis, MO, USA) was dissolved in dimethyl sulfoxide. Bacteria-derived recombinant mouse RANKL (R&D Systems, Minneapolis, MN, USA). The Diagnostic Acid Phosphatase kit (Sigma-Aldrich) for tartrate-resistant acid phosphatase (TRAP) staining. The Cell Counting Kit-8 (CCK8) (Dojindo Molecular Technology, Tokyo, Japan).

### 4.2. Animals and Study Design

The animal protocol for this study was approved by the Animal Care and Experiment Committee of the Ninth People’s Hospital affiliated to the School of Medicine, Shanghai Jiao Tong University (approval No.HKDL(2013)51). Twenty-four healthy 8-week-old C57BL/J6 female mice were assigned to 4 groups of 6 mice each randomly: phosphate-buffered saline (PBS) control (sham), OVX + ligature + PBS (vehicle), and OVX + ligature + low or high (2 or 5 mg∙kg^−1^∙day^−1^, respectively) dose myricetin [13]. The myricetin and PBS were injected intraperitoneally (i.p.) every other day for 30 days. Referring to the methods described by Wu *et al.* [13], both low and high (2 or 5 mg∙kg^−1^∙day^−1^, respectively) dose myricetin, which were injected every other day for 14 days, have inhibitory bone loss in mouse model. According to the methods of Xu *et al.* [29], simvastatin, which was given (local or oral) for two months, could prevent alveolar bone loss in rat model of periodontitis. According to above studies and our experience, we try to set this dose and experimental period.

According to Kobayashi [18], the OVX mouse model exhibits severe alveolar bone loss (ABL) and is a suitable model of periodontitis; therefore, this model was selected for this study. The mice were bilaterally OVX or a sham operation was performed under anesthesia with 1.2% avertin solution (i.p.). No adverse effects or mortality occurred during the experiment and at the end the mice were euthanized, and the maxillae was fixed in 4% (*w*/*v*) paraformaldehyde for micro-CT analysis.

### 4.3. Micro-CT Scanning and Assessment of Alveolar Bone

The fixed maxillae were analyzed using a high-resolution micro-CT scanner (Skyscan 1072, Skyscan, Aartselaar, Belgium). The scanning protocol was set at an isometric resolution of 9 μm, and the X-ray energy settings were 80 kV and 80 μA. According to the methods of Xu *et al.* [29], scans were reconstructed to generate three-dimensional models and the region of interest (ROI) was a cuboidal bone body that encompassed the roots. The ROI length extended from the most mesial to the most distal aspects of the upper first molar (M1) and upper second molar (M2) roots, respectively. Its width extended from the most buccal to the most palatal aspect of the M1 or M2 roots, and the height extended from the most apical aspect of M1 or M2 root to the most coronal part of the alveolar bone crest (ABC). The BV/TV, Tb.Th, Tb.Sp and Tb.N of each sample were measured as reported previously [42].

Alveolar bone loss (ABL), represented by the linear distance of the cemento-enamel junction (CEJ) to the ABC, was measured at two points on each tooth (M1), which were the mesiobuccal (MB) and distobuccal (DB) [29]. A higher ABL value implies a more severe bone loss.

### 4.4. Histological and Histomorphometric Analysis

The maxillae were decalcified in 10% (*w*/*v*) ethylenediaminetetraacetic acid (EDTA, pH 7.4) for 3 weeks, and embedded in paraffin, after which the histological sections were prepared. After H&E and TRAP staining, the specimens were examined and photographed under a high-quality microscope (Nikon eclipse 90i, Tokyo, Japan).

### 4.5. Osteoclasts Culture

RAW264.7 cells were cultured in complete α-MEM, which contained 10% (*v*/*v*) heat inactivated FBS, 100 U/mL penicillin/streptomycin, and 2 mM l-glutamine. The cell cultures were maintained in a humid environment with 5% CO_2_ at 37 °C.

### 4.6. Cell Viability and Cytotoxicity Assay

A CCK8 assay was used to determine the effect of myricetin on RAW264.7 cells viability. Briefly, RAW264.7 cells were plated in 96-well plates at a density of 1 × 10^4^ cells/well and cultured in complete α-MEM, which contained different concentrations of myricetin (0, 12.5, 25, 50, 100, 200, 400, and 800 μM) for 96 h. Then, 10 mL CCK-8 was added to each well, and the plates were incubated at 37 °C for an additional 2 h. The optical density (OD) was measured using an ELX800 absorbance microplate reader (Bio-Tek, Winooski, VT, USA) at a wavelength of 450 nm (650 nm reference). The cell viability was calculated relative to the control using the following formula: (experimental group OD − zeroing OD)/(control group OD − zeroing OD).

The GraphPad Prism version 5.0c (San Diego, CA, USA) was used to calculate the half-maximal inhibitory concentration (IC_50_).

### 4.7. TRAP Staining

RAW264.7 cells were seeded in a 96-well plate at a density of 8 × 10^3^ cells/well with complete α-MEM, myricetin (0, 10, or 50 μM) and RANKL (50 ng/mL). The culture medium was replaced every 2 days until osteoclasts formed on day 7. The Diagnostic Acid Phosphatase kit was used for TRAP staining of cells, which were fixed with 4% (*w*/*v*) paraformaldehyde for 20 min. TRAP-positive cells, which had more than 3 nuclei, were counted as osteoclast-like (OCL) cells. Three wells were randomly selected for each group for further analysis. The average number of OCL cells was calculated.

### 4.8. Resorption Pit Assay

RAW264.7 cells (2.4 × 10^4^ cells/cm^2^) were seeded onto bovine bone slices in complete α-MEM and stimulated with myricetin (0, 10, and 50 μM), and RANKL (50 ng/mL) until OCL cells were observed on Day 7. The OCL cells were removed from the bone slices using mechanical agitation and sonication. The scanning electron microscope (SEM, FEI Quanta 250, Hillsboro, OR, USA) was used to observe the resorption pits. Three view fields were randomly selected for each bone slice for further analysis. At least three similar independent experiments were repeated. The Image J software (National Institutes of Health, Bethesda, MD, USA) was used to quantify the percentage of resorbed bone surface area.

### 4.9. Statistical Analyses

Values are expressed as the mean ± standard deviation (SD). The independent replicates of each experiment were conducted at least thrice. The Student’s *t*-test was used to determine the statistically significant differences between the groups with the statistical package for the social sciences (SPSS) 17.0 software (SPSS Inc., Chicago, IL, USA). Differences were considered significant at *p* < 0.05 or *p* < 0.017 (Bonferroni correction for *p* value).

## 5. Conclusions

In summary, our study demonstrated that myricetin has a positive effect on OVX mice with experimentally induced periodontitis. Furthermore, the study also showed that myricetin inhibits osteoclast formation, and, in particular, at high doses, shows a superior effect in preventing alveolar bone loss in the mouse model. Taken together, our study strongly suggests that myricetin may be a potentially useful agent in the treatment of periodontitis and osteoporosis.
